# Autogenous Tooth Transplantation as a Treatment Option

**DOI:** 10.5005/jp-journals-10005-1142

**Published:** 2012-02-24

**Authors:** Ankita Chugh, Rashmi Aggarwal, Vinay Kumar Chugh, Puneet Wadhwa, Munish Kohli

**Affiliations:** Reader, Department of Oral and Maxillofacial Surgery, Saraswati Dental College and Hospital, Lucknow, Uttar Pradesh, India, e-mail: ankitamody@gmail.com; Reader, Department of Oral and Maxillofacial Surgery, Chandra Dental College, Barabanki, Uttar Pradesh, India; Assistant Professor, Department of Orthodontics, Faculty of Dental Sciences, CSM Medical University, Lucknow, Uttar Pradesh, India; DMD Student, School of Dental Medicine, Boston University, MA USA; Professor and Head, Department of Oral and Maxillofacial Surgery, Saraswati Dental College, Lucknow, Uttar Pradesh, India

**Keywords:** Autotransplant, Tooth transplantation

## Abstract

Autogenous tooth transplantation is the surgical movement of a tooth from one location in the mouth to another in the same individual. Though done for years but it has achieved variable success rates. Although the indications for autotransplantation are narrow, careful patient selection coupled with an appropriate technique can lead to exceptional esthetic and functional results. This article discusses the reviews of previous works done and highlights the criteria and factors influencing the success of autotransplant along with reports of two cases of transplantation of impacted and malposed canine.

**How to cite this article:** Chugh A, Aggarwal R, Chugh VK, Wadhwa P, Kohli M. Autogenous Tooth Transplantation as a Treatment Option. Int J Clin Pediatr Dent 2012;5(1):87-92.

## INTRODUCTION

Medical science today has progressed to an era where any nonfunctional kidney, heart, lungs can be replaced by transplantation of another functional organ. So replacing one damaged or missing tooth by another healthy functional tooth from the same oral cavity of 32 counterparts is surely promising. This surgical movement of a embedded, impacted or erupted vital or endodontically treated tooth from its original location in the mouth to another site in same individual is termed as autogenous tooth transplantation or autotransplantation.^1^ The new location may be a fresh extraction socket after extraction of a nonrestorable tooth, or an artificially-drilled socket on an edentulous alveolar ridge.^2^

Although tooth transplantation began earlier, it was not very common in our routine dental practice. The earliest reports of tooth transplantation involve slaves in ancient Egypt who were forced to give their teeth to their pharaohs.^3^However, this autotransplantation of a tooth from one individual to another was eventually abandoned because of problems of histocompatibility and replaced with autotransplantation.

Autotransplantation was first well-documented in 1954 by ML Hale.^1^ The major principles of his technique are still followed today. While there are many reasons for autotransplanting teeth, tooth loss as a result of dental caries is the most common indication. Other conditions in which transplantation can be considered include tooth agenesis especially of premolars and lateral incisors, traumatic tooth loss, atopic eruption of canines, root resorption, large endodontic lesions, cervical root fractures, localized juvenile periodontitis as well as other pathologies.^3,4^ We here are presenting two cases of autotransplantation of canines one impacted and other malposed to their normal position.

## CASE REPORTS

### Case 1

A 24-year-old lady reported to our department with a chief complaint of missing teeth. On examination, she got her 53 which was retained extracted 2 months ago creating an empty visible space. Her 13 was malposed over labial region of 14. After radiographic workup an initial socket based on estimated dimensions of permanent canine was drilled in the edentulous space and then 13 was extracted atraumatically. Then taking 13 as template socket was modified and in between intervals of socket modification tooth was replaced back in its existing socket. Copious irrigation was maintained while drilling of socket to avoid heat generation and minimum handling of root of the tooth was tried. Good snugly fit of the tooth was achieved. After getting tooth in proper arch alignment and good incisal clearance it was stabilized with arch bar for a period of 2 weeks. Root canal treatment was started on 12th day and completed with adequate seal. Tooth is being followed for 1 year now and shows good stability (Figs 1A to I).

### Case 2

A 16-year-old female patient was referred from orthodontics department for impacted canine. Her deciduous lower right canine was still present and based on radiographic analysis it was planned to extract her deciduous tooth and simultaneously remove her labially impacted canine atraumatically and enlarge the deciduous socket to accommodate the permanent counterpart. Her orthodontic treatment was almost complete except this part. A lower trapezoidal flap extending from premolar to premolar was raised and impacted canine was exposed and adequate bone removal was done to atrumatically remove the tooth. Deciduous tooth was extracted and then keeping permanent as template socket was modified by drilling around it. After an adequate fit of tooth was achieved it was stabilized using arch bar for 3 weeks. Arch bar was removed and replaced with their retention appliance (Figs 2A to F).

**Fig. 1A:**
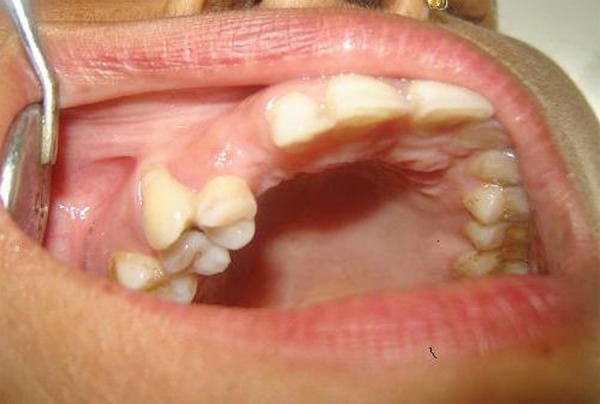
Preoperative (case 1)

**Fig. 1B:**
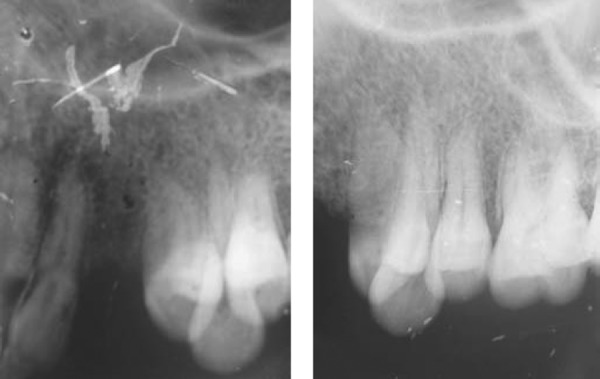
Preoperative IOPA

**Fig. 1C:**
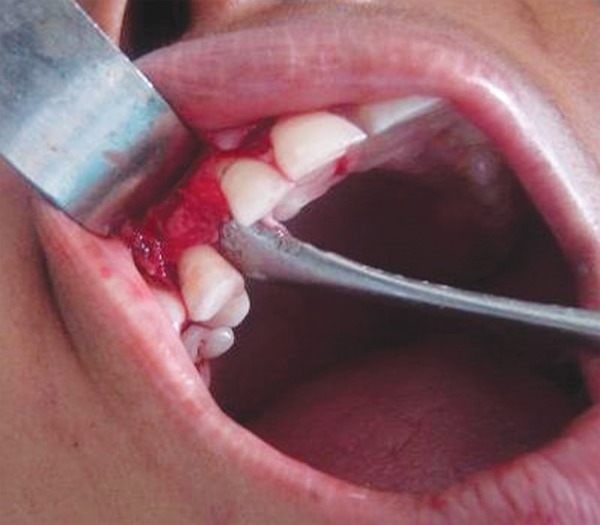
Site for artificial drilling exposed

**Fig. 1D:**
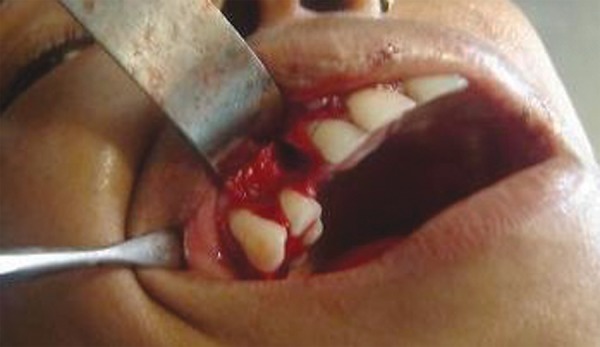
Initial drilling of socket

**Fig. 1E:**
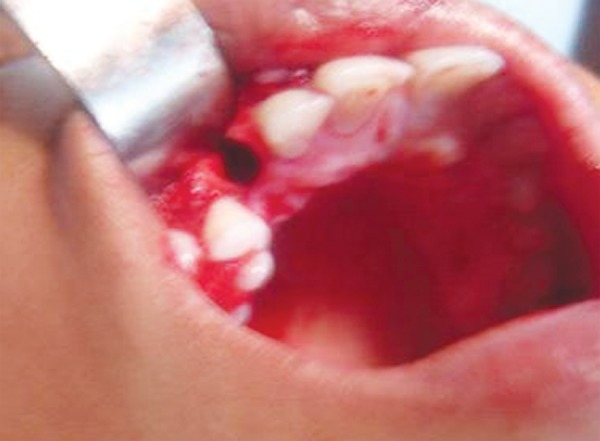
Socket manipulated according to malposed 13

**Fig. 1F:**
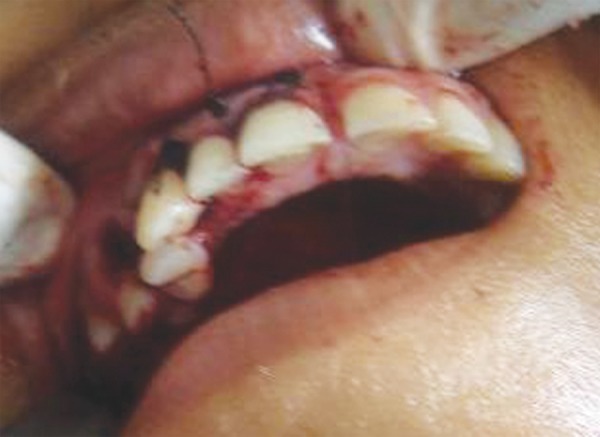
Malposed 13 transplanted

**Fig. 1G:**
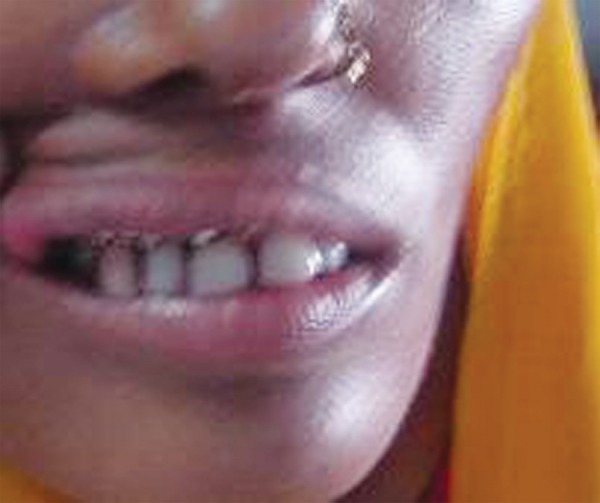
Malposed 13 splinted

**Fig. 1H:**
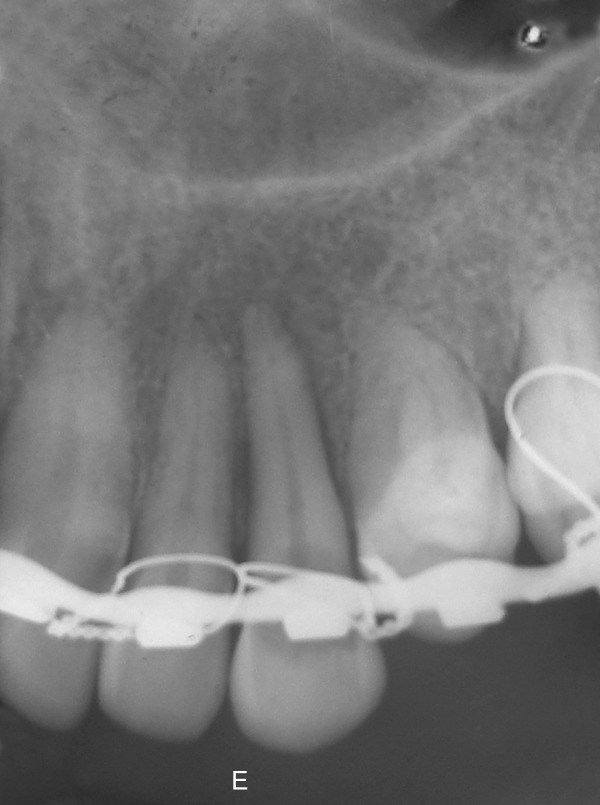
Immediate postoperative IOPA

**Fig.1I:**
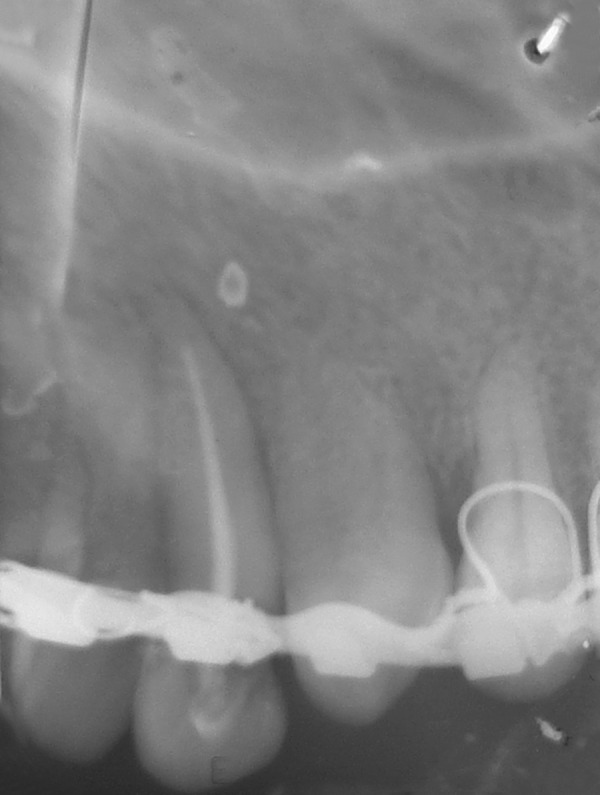
One month postoperative

## DISCUSSION

The understanding of the healing process of a transplanted tooth is imperative to its success. The preservation of favorable periodontal ligament (PDL) on the donor tooth is the critical factor for success. Reattachment occurs in about 2 weeks after autotransplantation between the PDL connective tissues of the donor root surface and the wall of recipient socket. When the damaged PDL surface is small, the healing can be achieved by cemental healing. However, when the damaged PDL surface is large, some of the root surface will be resorbed followed by apposition of bone rather than dentine, thus root resorption will ensure.^2^

Freshly extracted recipient sockets demonstrate higher success rate compared to artificially drilled ones due to contributions of the progenitors PDL cells on the recipient fresh extraction sockets. For proper differentiation of the PDL cells it is important to minimize inflammation. Inflammation will be minimized when the transplanted tooth is sealed with tight suturing by trimming and suturing of the gingival cuff around the tooth to prevent ingress of infective agents.^2^ It is also important to minimize inflammatory pulpal response from the transplanted tooth. For fully developed donor teeth, root canal treatment should be initiated 2 weeks after transplantation. The interim period of 2 weeks is chosen to minimize trauma to the PDL in the initial reattachment healing phase, yet further delay will increase the chance of complication of inflammatory resorption secondary to pulpal infection. In the case of donor tooth with incomplete root formation, the preservation of the apical Hertwig’s epithelial sheath is important to ensure pulpal regeneration and root maturation and eruption and therefore saving subsequent root canal procedures.

Successful transplantation depends on specific requirements of the patient, the donor tooth and the recipient site.^1^

**Fig. 2A:**
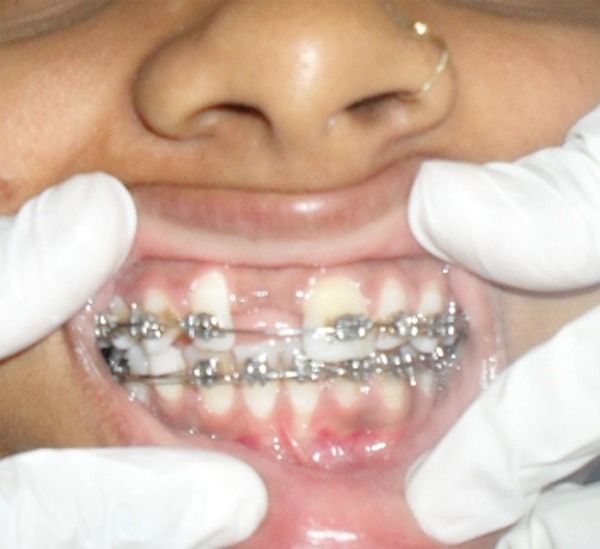
Preoperative (case 2)

**Fig. 2B:**
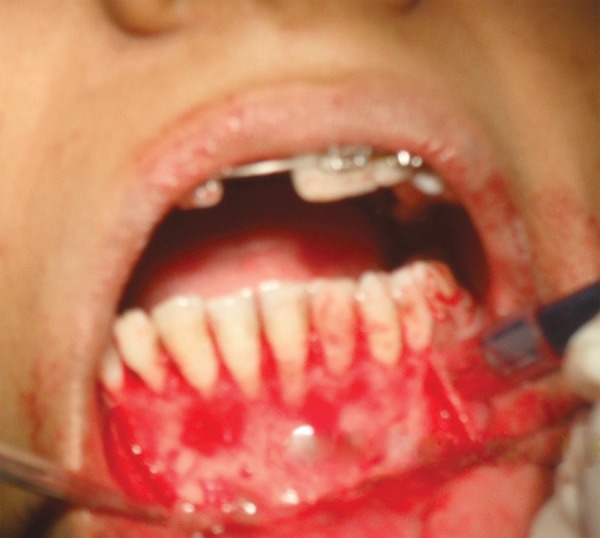
Flap raised

**Fig. 2C:**
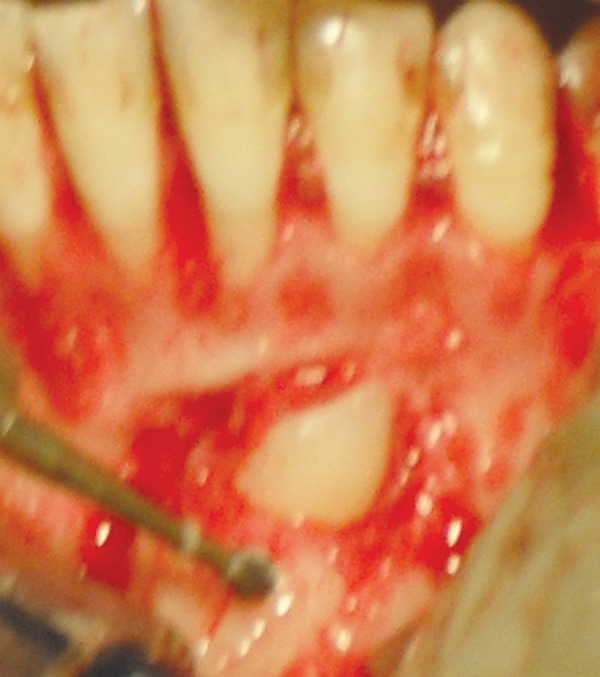
Impacted canine exposed

### Candidate Criteria

Candidates must be in good health with acceptable level of oral hygiene, able to follow postoperative instructions and available for follow-up visits. Most importantly, the patients must have a suitable recipient site and donor tooth.

**Fig. 2D:**
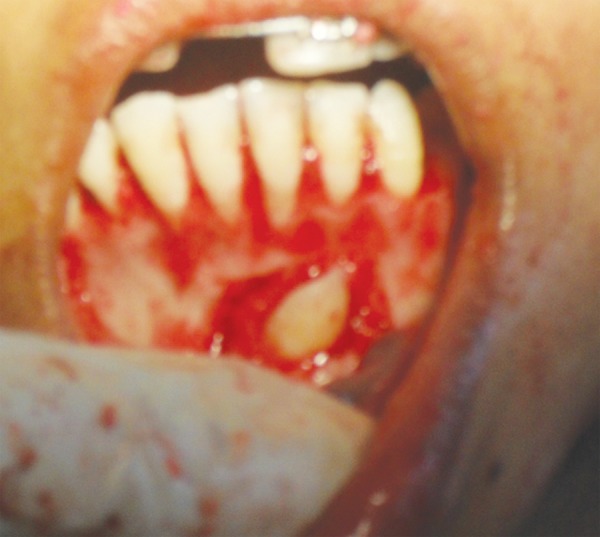
Impacted canine luxated

**Fig. 2E:**
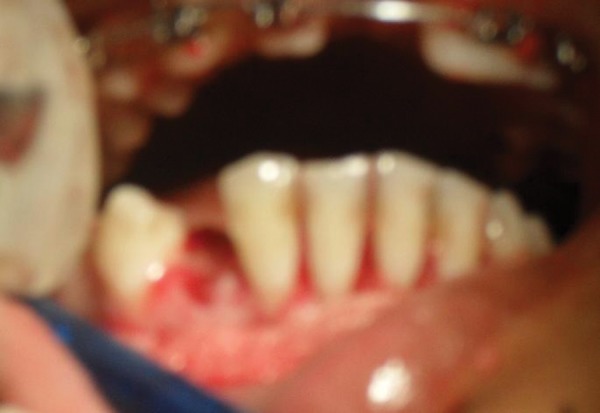
Deciduous canine extracted

**Fig. 2F:**
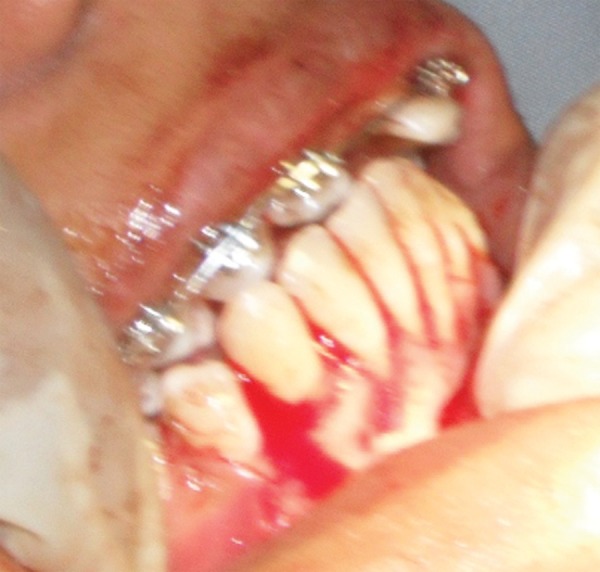
Impacted canine transplanted in final position

### Recipient Site Criteria

The most important criteria for success involving the recipient site is adequacy of sufficient alveolar bone support in all dimensions with adequate attached keratinized tissue to allow for stabilization of the transplanted tooth. In addition, the recipient site should be free from acute infection and chronic inflammation.^12^

### Donor Tooth Criteria

Most commonly used ones are premolars, canines, incisors and third molars. Initially reports concentrated on autotransplantation of either third molars or impacted canines, however in the late 1950s reports began to appear regarding the autotransplantation of other teeth.^5^

The donor tooth should be in such a position that extraction will be as atraumatic as possible. Abnormal root morphology, which makes removal exceedingly difficult is contraindicated for this surgery. Teeth with either open or closed apices may be donors; however, the most predictable results are obtained with teeth having between one-half to two-thirds completed root development.

After transplantation in cases where stability of transplant is in doubt then bonded wires may be used for 1 to 2 weeks. According to Pogrel^6^ rigid splinting has adverse effects on periodontal and pulpal healing. Flexible splinting for 7 to 10 days is more appropriate as it allows functional movement as this movement is said to stimulate periodontal ligament cellular activity and bone repair.

The classical autotransplantation technique involves the extraction of the donor tooth and preparation of the recipient site, using the donor tooth as a template. To minimize the extra-alveolar period, the donor site needs to be prepared so that further remodelling of the socket is not required once the graft is extracted. The use of surgical templates has been proposed to aid in socket preparation. Kugelberg et al reported the use of a selection of previously extracted and sterilized teeth as surgical templates.^7^ These previously extracted teeth would be sized against the preoperative radiograph of the graft tooth, and then the closest match would be used to prepare the donor socket. Refinements to this technique have included casting models of the extracted teeth in cobalt-chrome to aid effective sterilization. However, the significant disadvantage of this technique is that the surgical template is only a crude estimate based on a magnified two-dimensional radiograph. Surgical templates can be produced from the three-dimensional data provided by the CBCT, by using a technique called rapid three- dimensional prototyping. It aims to create an accurate physical three-dimensional model quickly from computerized data. It is hoped that the application of 3D prototyping will enhance the success of autotransplantation by making the technique less operator-sensitive and dramatically reduce extraoral time for the transplant.^8^

The available literature reports excellent success rates following tooth transplantation when the appropriate protocol is followed. Andreasen^9^ found 95 and 98% long- term survival rates for incomplete and complete root formation of 370 transplanted premolars observed over 13 years. Lundberg and Isaksson^10^ had success in 94 and 84% of cases for open and closed apices respectively in 278 autotransplanted teeth over 5 years. Kugelberg et al^11^ followed 23 immature and 22 mature teeth in 40 patients for up to 4 years, and reported success rates of 96 and 82% respectively. Czochrowska et al^12^ reported on long-term success of autotransplanted teeth. They followed up 30 autotransplanted immature teeth, with a follow-up period of 17 to 41 years (mean 26.4 years), and reported success and survival rates of 90 and 72% respectively.

However, the reports of earlier done studies were variable and did not show such high success rates. Schwartz and others^13^ showed success rates of only 76.2% at 5 years and 59.6% at 10 years. Similarly Pogrel^6^ found that his success rate for 416 autotransplanted teeth was 72%.

The factors that lead to success have been extensively investigated. The most significant determinant for survival of the transplant is the continued vitality of the periodontal membrane. In cases where the periodontal ligament is traumatized during transplantation, external root resorption and ankylosis is often noted. Schwartz^13^ tried to link the loss of the graft to specific prognostic factors and found that success rates were highest when donor teeth were premolars, had one-half to two-thirds root development, and experienced minimal trauma and limited extraoral time during surgery. Kristerson and Lagerström^4^ reported that all the teeth that failed in their study had reports of difficulties in the surgical removal from the donor sites in the patient records. In the study by Andreasen et al,^7^ it was found that length of extra-alveolar period was significantly related to future development of pulpal necrosis in the transplanted tooth. Here, it was found that of graft teeth stored extra-alveolar for <1 minute, 7 out of 102 (7%), developed pulp necrosis, whilst in teeth stored for >1 minute, 51 out of 258 (20%), would go on to develop pulp necrosis. Some specific parameters have been used to measure the health of the surviving transplant. These parameters include marginal periodontal attachment, mobility, pain, root resorption, root development, sensitivity to percussion, gingival pocket depth, presence of gingivitis and fistulae.^1,3-5^

The most common cause of failure of the autotransplant is chronic root resorption. The causes of tooth loss reported following transplantation from most common to least common are inflammatory resorption, replacement resorption (ankylosis), marginal periodontitis, apical periodontitis, caries and trauma. Inflammatory resorption may become evident after 3 or 4 weeks, while replacement resorption may not become evident until 3 or 4 months after transplantation. The incidence of both types of resorption can be decreased with atraumatic extraction of the donor tooth and immediate transfer to the recipient site to minimize the risk of injury to the periodontal ligament.^1^

To assess the usefulness of this technique its comparison with implantology is inevitable. Implantology has become popular in recent years in terms of predictability in both success rate and esthetic result. However osseointegrated implants are contraindicated in the growing child because they behave like an ankylosed tooth and do not maintain their position in the arch as further growth of the maxilla occurs, becoming increasingly submerged. The beauty of transplanted teeth is that they are biological and able to erupt in harmony with adjacent teeth and growing jaws. Autotransplantation if successful ensures that alveolar bone is maintained due to physiological stimulation of the periodontal ligament.

Cost-effectiveness is another obvious advantage of this procedure which enables the utilization of a tooth that is hitherto nonfunctional. The main disadvantages are surgical involvements, technique sensitivity, relatively low versatility in their applications (e.g. tooth and space size discrepancy) and more importantly low predictability in results compared to conventional prosthetic restoration like implants, bridge and dentures.

## CONCLUSION

The science of autotransplantation has progressed and studies demonstrate that it is a viable option for tooth replacement for carefully selected patients. There is obvious limitation in terms of versatility in the application of transplantation *vs *implantation because of size and morphology of donor tooth being the major constraint. It is also more technique sensitive. However, our experience in autotransplantation demonstrates that it is a viable treatment alternative especially in growing adolescents as it provides a biological and economical treatment alternative for tooth replacement.
